# Biomarkers in gastroesophageal cancer 2025: an updated consensus statement by the Spanish Society of Medical Oncology (SEOM) and the Spanish Society of Pathology (SEAP)

**DOI:** 10.1007/s12094-025-03865-6

**Published:** 2025-03-12

**Authors:** Maria Alsina Maqueda, Ana Teijo Quintáns, Miriam Cuatrecasas, Maria Jesús Fernández Aceñero, Ana Fernández Montes, Carlos Gómez Martín, Paula Jiménez Fonseca, Carolina Martínez Ciarpaglini, Fernando Rivera Herrero, Mar Iglesias Coma

**Affiliations:** 1 Medical Oncology Department, Unidad de Oncología Médica Traslacional, Hospital Universitario de Navarra, Navarrabiomed -Instituto de Investigación Sanitaria de Navarra, Pamplona, Spain; 2 Pathology Department, Gastrointestinal and Neuroendocrine Tumors Research Group, Hospital Universitario 12 de Octubre, Research Institute (Imas12), Madrid, Spain; 3 Pathology Department, Hospital Clinic de Barcelona, Biomedical Research Institute IDIBAPS, University of Barcelona, Barcelona, Spain; 4 Department of Legal Medicine, Psychiatry and Pathology, Surgical Pathology Department, Hospital Clínico Universitario San Carlos, nstituto de Investigación Sanitaria Clínico San Carlos (IdISSC), Universidad Complutense, Madrid, Spain; 5 Madrid, Spain; 6 Medical Oncology Department, Complexo Hospitalario Universitario de Ourense, Ourense, Spain; 7 Gastrointestinal Cancer and Early Clinical-Translational Research Units, Medical Oncology Division, 12 de Octubre University Hospital, Madrid, Spain; 8 Department of Medical Oncology, Hospital Universitario Central de Asturias, ISPA, Oviedo, Spain; 9 Pathology Department, Hospital Clínico Universitario de Valencia, Biomedical Research Institute INCLIVA, University of Valencia, Valencia, Spain; 10 Medical Oncology Department, Hospital Universitario Marqués de Valdecilla, IDIVAL, Santander, Spain; 11 Pathology Department, Hospital del Mar, Pompeu Fabra University, Hospital del Mar Research Institute, Barcelona, Spain

**Keywords:** Gastroesophageal carcinoma, Biomarkers, PD-L1, Claudin 18.2, HER2, MSI/dMMR

## Abstract

Gastroesophageal carcinomas, including gastroesophageal adenocarcinoma (GEA) and esophageal squamous cell carcinoma (ESCC), pose a global health challenge due to their heterogeneity. The approach to diagnosis and treatment should first differentiate between GEA and ESCC. Over the past decade, therapies for metastatic or advanced GEA/ESCC have expanded, with several new therapeutic targets alongside trastuzumab for metastatic HER2-positive GEA. Four key biomarkers are essential for targeted therapy: HER2 overexpression/amplification, deficient mismatch repair/microsatellite instability (dMMR/MSI), PD-L1, and Claudin18.2 expression. Immunohistochemistry is the recommended method for these biomarkers evaluation. In addition, the assessment of biomarkers like FGFR2b is likely to become routine in the near future. Experts from the Spanish Society of Pathology (SEAP) and the Spanish Society of Medical Oncology (SEOM) have formed a consensus to optimize biomarker detection and usage in clinical practice. Their recommendations aim to improve personalized treatment strategies for GEA and ESCC patients, integrating new diagnostic insights into routine care.

## Introduction

Gastroesophageal carcinomas comprise a heterogeneous group of tumors that represent a significant health problem.

Esophageal cancer is the 11th most common tumor worldwide and the 7th cancer in terms of mortality [1]. In Spain, 2269 new cases will be diagnosed in 2024, representing the 19th leading cause of mortality in 2020 (1847 deaths) [2]. There are two main histological subtypes of esophageal cancer: esophageal squamous cell carcinoma (ESCC) and esophageal adenocarcinoma (EAC). Although ESCC represents approximately 90% of esophageal cancer cases worldwide, the incidence of EAC is increasing and has surpassed the incidence rate of ESCC in Europe and North America [3]. These two histological variants are associated with different etiopathogenic factors. ESCC is linked to excessive alcohol consumption and smoking, and to nutritional deficiencies and nitrosamines exposition in developed countries. In contrast, EAC is associated with gastroesophageal reflux and obesity [4].

On the other hand, gastric cancer (GC) is the 5th leading cause of cancer worldwide [1], with 6868 new cases expected in Spain in 2024 representing the 8th leading cause of mortality in 2020 (4757 deaths) [2]. GC incidence varies significantly geographically, with higher rates in Eastern Asia, Eastern Europe, and South America while Western Europe and the USA have seen a gradual decline in incidence over the last 60 years. This decline has been attributed to a decreased prevalence of *H. pylori* and improved food preservation and storage. However, there has been a slight increase in cardia and gastroesophageal junction cancers (GEJC), probably related to similar causes to EAC. Recent epidemiologic data also show an increase in the incidence of gastroesophageal cancer in young adults (aged < 50 years), mainly in high-income countries. Contributing factors include environmental influences, autoimmune gastritis, and dysbiosis of the gastric microbiome related to the increased use of antibiotics and acid suppressants [5]. Finally, familial aggregation can explain ∼ 10% of GC cases, while up to 3% occur within hereditary genetic syndromes, like hereditary diffuse gastric cancer (HDGC), familial intestinal GC (FIGC), and the gastric adenocarcinoma and proximal polyposis of the stomach (GAPPS). GC can also occur in other inherited cancer disorders, including familial adenomatous polyposis, MUTYH-associated polyposis, Peutz–Jeghers, juvenile polyposis, Lynch, Li-Fraumeni, Cowden, and hereditary breast and ovarian cancer syndromes [6, 7].

### Gastroesophageal adenocarcinoma (GEA)

GC was historically viewed as distinct from EAC, and GEJC was classified as either esophageal or gastric cancer, due to the duality of the tumor location. In 2014, The Cancer Genome Atlas Program (TCGA) proposed considering all gastroesophageal adenocarcinomas (GEAs) as one disease, but with deep recognition of the relevant heterogeneity, acknowledging their molecular diversity [8]. The World Health Organization (WHO) classified GEA into six histological subtypes [9]: tubular, papillary, poorly cohesive (including signet ring cell), mucinous, carcinoma with lymphoid stroma, and mixed. TCGA analysis, however, defines four molecular subtypes: Epstein–Barr virus (EBV), microsatellite instability (MSI), chromosomal instability (CIN) and genomically stable (GS) [8, 10]. The CIN subtype is the most frequent (50%), predominantly found in upper stomach characterized by intestinal (tubular/papillary) histology. Key features include enrichment for *TP53* mutations, amplification of receptor tyrosine kinases (RTK) genes (*EGFR*, *ERBB2 (HER2)*, *ERBB3*, *FGFR2*, *JAK2* and *MET*), *KRAS* or *NRAS* mutations, alterations in cell-cycle mediators and *VEGFA* amplification, some of which can be targeted. EBV carcinomas primarily occur in the proximal stomach in younger patients, typically showing dense lymphocyte infiltration and widespread expression of immune-checkpoint proteins, such as programmed cell death ligand 1 (PD-L1), highlighting the generally high immunogenicity of this subtype. EBV carcinomas also usually display a CpG island methylator phenotype, with recurrent mutations in *PIK3CA*, *ARID1A*, and *BCOR*. The MSI subtype arises from impaired DNA mismatch repair (MMR) function, typically due to MLH1 promoter methylation or mutations in MMR-related genes. Finally, the GS subtype, which often arises in the distal stomach, is enriched by poorly cohesive (diffuse) histology, and is associated with younger age. It presents mutations in *CDH1* or *RHOA*, as well as intrachromosomal translocation between *CLD18* and *ARHGAP26/6*, which are linked to epithelial-to-mesenchymal transition (EMT). The recognition of the intrinsic heterogeneity of EAC is crucial for a good precision oncology. GEA exhibit intertumoral and intratumoral heterogeneity in tumor morphology and molecular characteristics, with variability between patients (intertumoral heterogeneity) and within the same tumor (intratumoral heterogeneity) [11]. This includes spatial heterogeneity across tumor regions and temporal heterogeneity during disease progression, often related to resistance mechanisms against targeted therapies during primary, recurrent, and metastatic stages.

## Treatment of locally advanced unresectable or metastatic GEA

**Fig. 1:**
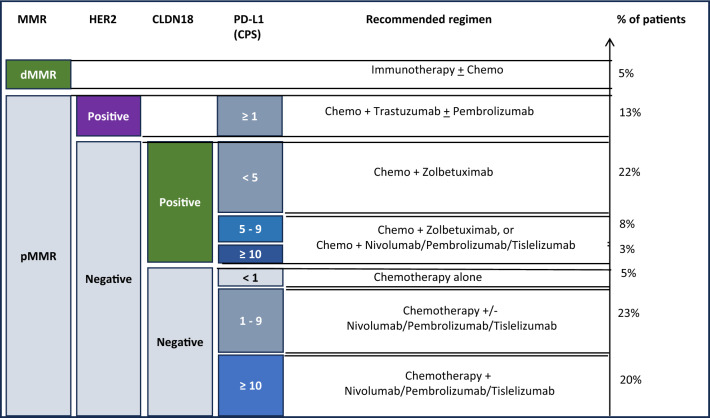
Proposed algorithm for treatment of advanced GEA patients according to biomarker status. GEA patients are treated according to biomarkers evaluation, including MMR, HER2, CLDN18.2 and the score for PD-L1. Target therapies, including immunotherapy, are combined with chemotherapy according to the expression of the indicated biomarkers (adapted from 10.1016/j.esmogo.2024.100086)

Cytotoxic chemotherapy including fluoropyrimidines, platinum compounds, taxanes, and irinotecan, remains the cornerstone of advanced GEA treatment. Fluoropyrimidine and platinum combination therapy is the standard first-line treatment, with oxaliplatin being as effective as cisplatin [6]. In patients unsuitable for intensive chemotherapy, a modified two-drug regimen offers better tolerability without compromising outcomes [12]. Taxanes and irinotecan are used in second-line treatment. In addition, targeted therapies have demonstrated improved survival for GEA patients, depending on associated biomarker expression (Fig. 1).

### Anti-HER2 therapy

In the first-line treatment of HER2-positive advanced GC and GEJC, patients receiving platinum-based chemotherapy and the anti-HER2 antibody trastuzumab showed improved objective responses and survival rates (median overall survival [mOS] 16.0 vs. 11.8 months; hazard ratio [HR] 0.65 [95% CI, 0.51–0.83]) [13]. T-Dxd has been approved by the European Medicines Agency (EMA) in the refractory setting based on a single-arm phase 2 study (41.8% response rate [95% CI, 30.8‒53.4]) [14]. Furthermore, combining HER2-targeted therapies with immunotherapy, such as with pembrolizumab, has significantly improved survival in GEA patients with PD-L1 combined positive score (CPS) ≥ 1 (mOS 20.1 vs. 16.8 months; HR 0.80 [95% CI, 0.67–0.94]; *p* = 0.0040) and received EMA approval [15] (Fig. 1). Novel antibody–drug conjugates (ADCs) and more potent antibodies, such as margetuximab, tucatinib, and zanidatamab, are under development with promising results.

### Immunotherapy

Immunotherapy has significantly improved GEA treatment, particularly for dMMR/MSI and high PD-L1 tumors. Anti-PD1 antibodies, nivolumab, pembrolizumab and tislelizumab, have been evaluated with chemotherapy in the first-line setting. Nivolumab showed benefit in GEA patients with PD-L1 combined positive score (CPS) ≥ 5 (mOS 14.4 vs. 11.1 months; HR 0.70 [95% CI 0.61–0.81]) [16]. Pembrolizumab improved outcomes in GEA patients with PD-L1 CPS ≥ 10 (mOS 13.9 vs. 8.8 months; HR 0.57 [95% CI 0.43–0.75]; *p* < 0.0001), and CPS ≥ 1 (mOS 13.0 vs. 11.4 months; HR 0.74 [95% CI 0.43–0.75]; *p* < 0.0001) [17, 18]. Finally, tislelizumab also showed positive results, leading to EMA approval in November 2024 for GEA patients with tumor area positivity (TAP) ≥ 5% (mOS 17.2 vs. 12.6 months; HR 0.74 [95% CI, 0.59–0.94]; *p* 0.006) [19]. In Spain, nivolumab is approved for PD-L1 CPS ≥ 5 in GEA, and pembrolizumab is approved for PD-L1 CPS ≥ 10 in the first-line setting (Fig. 1). In advanced dMMR/MSI GEA, standard chemotherapy of fluoropyrimidine and platinum combinations has limited efficacy, while treatment with anti-PD1 ± anti-CTLA4 inhibitors show promise, especially for patients unable to undergo chemotherapy [20, 21]. However, these combinations remain unapproved due to the lack of registration studies, with only pembrolizumab approved in the refractory setting [22].

### Claudin-18.2 (CLDN18.2)

In two recently published phase III trials, zolbetuximab, a chimeric IgG1 antibody that targets CLDN18.2, has shown significant improvements in overall and disease-free survival when added to first-line chemotherapy in patients with high CLDN18.2 expression (≥ 75% of tumor cells). Final analysis of the two phase 3 trials revealed a mOS of 16.4 *vs*. 13.7 months (HR 0.77 [95% CI, 0.67–0.89]) [23–25]. EMA approved zolbetuximab in this setting in October 2024 (Fig. 1). In addition, new anti-CLDN18.2 drugs, including chimeric antigen receptor T cells and bispecific T-cell engagers, are being evaluated for enhanced targeting of CLDN18.2-positive tumor cells.

### Fibroblast growth factor receptor 2b (FGFR2b)

Bemarituzumab, a humanized IgG1 monoclonal antibody targeting FGFR2b, improved survival in FGFR2b-overexpressing GEA patients when combined with first-line chemotherapy in a randomized phase II trial [26]. Two-phase III trials are currently evaluating this strategy with/without immunotherapy.

### Antiangiogenics

In the second-line setting, the anti-angiogenic agent ramucirumab (anti-VEGFR2) when combined with paclitaxel improved OS compared to chemotherapy alone (mOS 9.6 vs. 7.4 months; HR 0.81 [95% CI 0.68–0.96]; *p* 0.17).[27]. However, anti-angiogenic drugs used in-first-line GC have not shown enough activity, as trials were designed for all-comers without biomarkers to guide selection.

## Current pathological considerations for locally advanced unresectable or metastatic GEA

### HER2

*ERBB2* (also commonly known as *HER2*) is a proto-oncogene on chromosome 17 encoding a 185-kDa RTK protein, member of the epidermal growth factor receptor family. Its phosphorylation triggers signaling pathways promoting cell division, proliferation, differentiation, and anti-apoptotic effects. In GEA, HER2 overexpression occurs in 8–20% of cases, more common in the distal esophagus, GEJ and proximal GC [13, 14, 28].

**Fig. 2:**
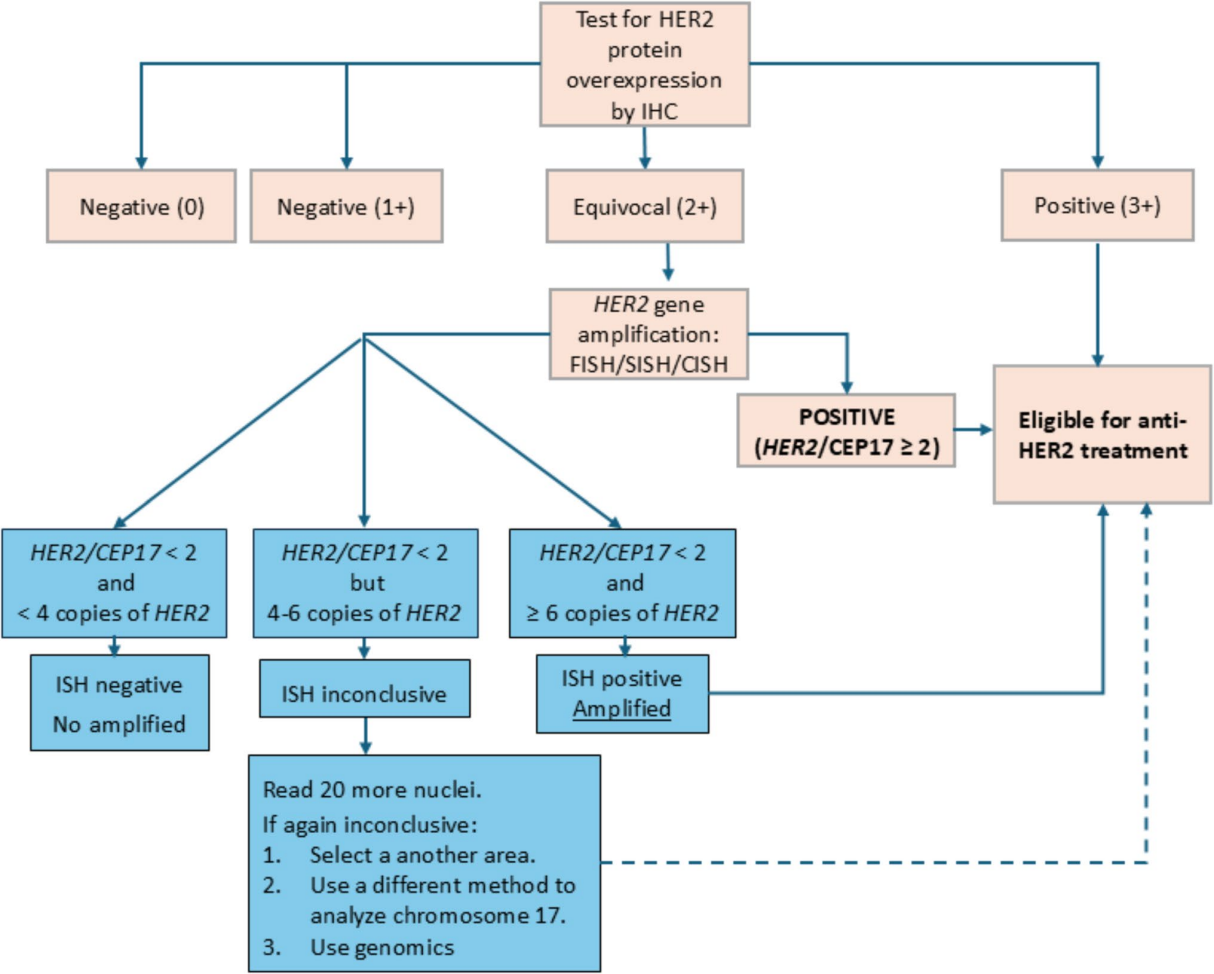
Test algorithm for human epidermal growth factor receptor 2 (HER2) detection. HER2 expression is used to select patients that respond to target therapy. The modified Hoffman scoring system assesses HER2 membrane staining, intensity, and percentage of immunoreactive cells by immunohistochemistry. In equivocal cases (IHC 2 +), FISH/SISH/CISH is performed. IHC, immunohistochemistry; FISH, fluorescent in situ hybridization; SISH, silver in situ hybridization; CISH, chromogenic in situ hybridization, CEP17, Chromosome 17

The modified Hoffman scoring system assesses HER2 membrane staining, intensity, and percentage of immunoreactive cells by immunohistochemistry (IHC) (Fig. 2). Gastric tumor cells often show incomplete HER2 membrane reactivity. The score differentiates between biopsy and surgical specimens. Surgical specimens with ≥ 10% of tumor cells with strong HER2 membrane expression or endoscopic biopsies with ≥ 5 cells showing strong expression are considered positive (3 +) [29]. Cases with weak to moderate intensity are considered equivocal (2 +), and faint or barely perceptible expression are considered negative (1 +) (Fig. 2). In equivocal cases (IHC 2 +), fluorescent/silver/chromogenic in situ hybridization (FISH/SISH/CISH) techniques should be performed to assess *HER2* gene amplification. FISH results are expressed as the ratio of *HER2* gene copies to the number of centromeres of chromosome 17 (CEP17) in the nucleus (HER2:CEP17). At least 20 tumor cells clustered in the area of highest protein expression should be counted. If the ratio is ≥ 2, or the ratio is < 2 but with more than 6 copies of *HER2*, the result is considered positive (amplified). If the ratio is < 2 with fewer than 4 copies*,* it is negative (not amplified). A ratio of < 2 and *HER2* copies between 4 and 6 is considered equivocal, and further analysis of 20 additional cells is recommended. If the result remains equivocal, a different area, a different sample, or an alternative technique (*e.g.*, molecular testing) may be used [29, 30] (Fig. 2). For the immunohistochemical determination of HER2, FDA and/or EMA -certified diagnostic kits should be used, and prior validation by the laboratory is recommended. The use of commercial kits requires strict adherence to the manufacturer’s instructions without modifications [28]. This method helps refine the diagnosis and guide treatment decisions for GEA patients based on HER2 status. The ASCO, NCCN, and ESMO guidelines recommend testing for HER2 status in all patients with advanced disease and suggest repeat biomarker testing in cases at progression in advanced or metastatic disease [6, 30–32].

### Programmed cell death ligand 1 (PD-L1) determined by CPS

PD-1 is an inhibitory checkpoint receptor protein expressed on cytotoxic T cells and other immune cells. By binding to its ligands PD-L1 and PD-L2, PD-1 maintains immune cell tolerance during peripheral and central immune regulation. Binding of PD-L1 to PD-1 inhibits T-cell receptor signaling. Some tumor cells express high levels of PD-L1 as an immune evasion mechanism, as the PD-1/PD-L1 interaction induces the inactivation of cytotoxic T cells and downregulation of immune responses [33].

The CPS is based on the following formula:

$${\text{CPS}} = \frac{{\text{Number of PD-L1 positive tumor cells}} + {{\text{Number of PD-L1 positive mononuclear inflammatory cells}}}}{{\text{Total tumor cells}}}\times {1}00 = 0 - {1}00.$$

Most GEA show PD-L1 expression in immune cells rather than tumor cells [34]. In GEA, approximately 35% of cases show CPS ≥ 10 [18], around 60% CPS ≥ 5 [35] and about 78% CPS ≥ 1, depending on the literature.

CPS results must be expressed as an absolute number ranging from 0 to 100, and terms “positive” or “negative” should be avoided (Dako manual). PD-L1 expression should only be evaluated in the invasive component of the lesion, excluding non-tumoral areas like necrosis, ulceration, granulation tissue, normal mucosa, gastritis, or dysplasia. Tumor cells exhibiting complete or incomplete membrane staining of any intensity are considered positive. Only surrounding mononucleated inflammatory cells within a 20X objective field must be assessed. Positive mononuclear inflammatory cells (lymphocytes and macrophages) may show cytoplasmic or membrane staining of any intensity. Other inflammatory cells such as plasma cells and neutrophils should be excluded (Dako manual).

Reliable PD-L1 IHC analysis requires formalin-fixed paraffin-embedded (FFPE) tumor samples with at least 100 viable tumor cells (Dako manual) [36]. The use of external controls and tissues, cell block and/or in-home tissue controls, should be included (Dako manual).

Although data are limited, studies indicate that 22C3, 28–8, and SP263 antibody clones might be interchangeable, particularly for CPS ≥ 5, with moderate to high interobserver concordance [37–40]. However, validation in specific clinical contexts is necessary.

### CLDN18.2

The claudin proteins, including CLDN18.2, are key components of the tight junction complex that regulates cell polarity and paracellular transport. CLDN18.2 is predominantly found in gastric mucosa and remains present in gastric tissues after malignant transformation, often becoming more exposed in malignant tissues [41]. Tumors are eligible for zolbetuximab treatment if they show moderate to strong (2/3 +) membranous staining (complete, basolateral, or lateral) for CLDN18.2 in ≥ 75% of tumor cells.

The Ventana CLDN18 (43–14A) assay, used on FFPE neoplastic tissues on BenchMark IHC instruments, can reliably detect CLDN18.2 expression with laboratory validation to ensuring consistency and reproducibility. This assay, approved for in vitro diagnostic (IVD), uses a companion diagnostic assay (Roche Ventana, Oro Valley, AZ, USA) that recognizes the C-terminus of CLDN18. This assay, therefore, recognizes both CLDN18.1 and CLDN18.2 isoforms, but provide a reliable evaluation of CLDN18.2 due to the minimal expression of CLDN18.1 isoform in the gastric setting. Moderate-to-strong membranous expression of CLDN18.2 in non-neoplastic gastric mucosa serves as a positive internal control [41].

In mixed-type tumors, it is important to prioritize CLDN18.2 determination in the poorly cohesive or diffuse components, as CLDN18.2 expression has been shown to be more associated with these histotypes [41]. Few studies have investigated the correlation of CLDN18.2 expression between primary tumors, synchronous lymph nodes, cytological effusion, and metastases. Therefore, stronger evidence is needed to confirm these indications [41].

### Mismatch-repair (MMR) proteins deficiency/MSI

Microsatellites are short, tandemly repeated DNA sequences (1–6 bases long) found throughout the genome, primarily near coding regions. The high level of MSI (MSI-H) phenotype results from mutations in repetitive sequences due to defects in the DNA MMR system, essential for monitoring and correcting DNA replication errors.

MSI-H can occur in inherited syndromes like Lynch syndrome, caused by mutations in *MMR* genes (*MLH1*, *MSH2*, *MSH6, PMS2*) or *EPCAM* genes, or from sporadic somatic mutations, often due to epigenetic silencing of *MLH1* through promoter hypermethylation, the leading mechanism of MMR loss in both sporadic and familial MSI-H GC cases [33].

IHC analysis of MMR proteins is a reliable, cost-effective method for MSI detection, offering excellent sensitivity and specificity when compared to polymerase chain reaction (PCR)-based testing [42, 43]. Four antibodies are recommended for the immunohistochemical evaluation of MMR proteins: MLH1, MSH2, MSH6, and PMS2 [44]. These four proteins are ubiquitously expressed in the cell nuclei. MMR deficiency should only be diagnosed when nuclear staining for any MMR protein is completely absent, supported by positive internal controls (*e.g.*, lymphocytes, stromal cells, and non-neoplastic epithelial cells), strongly suggesting MSI [45].

MMR proteins function as heterodimers, where MLH1 and MSH2 are obligate partners; their loss leads to degradation of its partner (PMS2 and MSH6, respectively). However, PMS2 and MSH6, secondary partners, can be compensated by other proteins (i.e., MSH3 and MLH3), retaining the expression of MLH1 and MSH2 [44, 46]. MLH1 and MSH2 alone do not identify cases with PMS2 or MSH6 abnormalities [44]. As a cost-effective approach, PMS2 and MSH6 can be used together for initial screening; however, any loss will prompt sequential IHC testing of the partner protein for confirmation. In cases of isolated PMS2 or MSH6 loss (below 5% of cases) or other atypical phenotypes, the MSI status should be confirmed by PCR or NGS [44, 47].

If IHC results are inconclusive, the ESMO Guidelines recommend PCR-based testing, as the gold standard, and an appropriate alternative to IHC. NGS also offers an alternative for simultaneous assessment of MSI status and tumor mutational burden in certain panels, providing a broader molecular profile [44, 46]**.**

## Pathological considerations for the near future

### Programmed cell death ligand 1 (PD-L1) as determined by TAP

TAP is another method for PD-L1 scoring based on visual assessment of stained tumor areas, providing a distinct approach to CPS for assessing PD-L1 expression. In clinical trials, the established positive cut-off is TAP ≥ 5% [19].

TAP is calculated by the percentage of tumor area stained for PD-L1 using the SP263 antibody (Ventana, Roche) including tumor cells and desmoplastic stroma, (membrane staining of any intensity) and tumor-associated immune cells (any intensity) [48].

Different studies compared three commercially available PD-L1 assays (22C3, 22-8, SP263) and two scoring algorithms (CPS and TAP). These studies reported similar staining patterns with the three PD-L1 assays and general agreement between CPS and TAP, indicating their possible comparability in assessing PD-L1 in tumors in the future [40].

### FGFR2b

FGFR is a transmembrane RTK that activates many downstream pathways, including MAPK and AKT, that are critical for cell growth, survival, and migration. Activation of *FGFR* signaling can result from gene amplification, activating mutations, or chromosomal translocations/fusions.

*FGFR2* amplification in GC is rare, occurring in 3–9% of cases. However, FGFR2b overexpression detected by IHC is more common, detected in 31–51% of cases. This suggests that IHC may be more useful than molecular detection methods [33]. Notably, bemarituzumab shows therapeutic benefit with FGFR2 overexpression, not amplification.

Positive FGFR2b expression was defined in a clinical trial as moderate to strong membranous and cytoplasmic staining in ≥ 10% of tumor cells. The final cut-off for targeted therapy needs to be evaluated in the future [26].

Interestingly, metastatic GC tissues and metastatic lymph nodes revealed a higher FGFR2b expression compared to paired primary tumors [33, 49]. Table 1 summarizes therapeutic approaches and pathological considerations for locally advanced unresectable or metastatic GEA.

**Table 1:** Bullet points for gastroesophageal adenocarcinoma

Therapeutic approaches for locally advanced unresectable or metastatic GEA
Trastuzumab combined with platinum-based therapy is the standard first-line treatment for HER2-positive advanced GEA The combination of trastuzumab and pembrolizumab was approved by EMA as first line therapy for HER2-positive, PD-L1 CPS ≥ 1 tumors, and Trastuzumab Deruxtecan as second line, for sustained HER2-positivity First line immunotherapy is used combined with standard chemotherapy in high PD-L1 GEA. Nivolumab (CPS ≥ 5) and pembrolizumab (CPS ≥ 10) are both good options in Spain, and tislelizumab has received recent EMA approval dMMR/MSI GEA patients need to be treated with immunotherapy approaches First-line Zolbetuximab combined with standard chemotherapy improves survival in tumors with ≥ 75% CLDN18.2 expression by IHC, and has been recently approved by EMA
Therapeutic approaches for locally advanced resectable GEA
Surgery alone is often sufficient for localized dMMR/MSI GEA without high-risk features; perioperative FLOT is preferred when high-risk features are present
Biomarkers used in GEA
MMR system and/or microsatellite status determination should be performed for all newly diagnosed GEA IHC is the preferred method for MMR testing, though PCR/NGS can also be used Biomarker determination for locally advanced unresectable or metastatic GEA include HER2 status, PD-L1 expression, and CLDN18.2 expression HER2 screening should be performed by immunohistochemistry ± HER2 in situ hybridization depending on the algorithm PD-L1 IHC CPS should be used, expressed as an absolute number. The terms positive/negative should be avoided. TAP is a novel score for PD-L1 assessment associated with emerging indications CLDN18.2 is an emerging therapeutic target evaluated by IHC

GEA, gastroesophageal adenocarcinoma; PD-L1, programmed death ligand 1; CPS, combined positive score; EMA, European Medicine Agency; dMMR/MSI, deficient mismatch repair protein/microsatellite instability; CLDN18.2, Claudin-18.2; IHC, immunohistochemistry; PCR, polymerase chain reaction; NGS, new generation sequencing

## Treatment of locally advanced resectable GEA

For locally advanced resectable GEA, the standard of care includes a perioperative FLOT chemotherapy (5-fluorouracil, leucovorin, oxaliplatin and docetaxel) and surgery with D2 lymphadenectomy [50].

A meta-analysis of phase III trials, including perioperative chemotherapy (MAGIC), adjuvant chemotherapy (CLASSIC, ITACA-S), and chemotherapy versus chemoradiotherapy (ARTIST), evaluated the prognostic and predictive role of the dMMR/MSI status in the perioperative setting [51]. GEA patients with dMMR/MSI tumors presented improved survival but did not benefit from perioperative/adjuvant chemotherapy. Nevertheless, the results of the randomized phase II DANTE trial with FLOT regimen suggested potential efficacy of taxanes-containing regimens in this setting [54].

### Perioperative immunotherapy

Two phase III trials, KEYNOTE-585 and MATTERHORN, evaluated the role of immune-checkpoint inhibitors (ICIs) in the perioperative setting, but did not select patients based on PD-L1 expression [52, 53]. Pembrolizumab added to a predominantly FOLFOX chemotherapy showed no survival benefit in GEA patients. However, adding durvalumab to the FLOT regimen increased pathologic complete response (pCR) rates, and also event free survival outcomes, according to a recent press-release [52].

In contrast, two non-randomized phase II studies demonstrated the efficacy of double immune inhibition with nivolumab-ipilimumab (GERCOR-NEONIPIGA) [55] and durvalumab-tremelimumab (INFINITY) [56] in dMMR/MSI tumors, with pCR rates of ≈ 60% and negligible relapse rates. These results highlight the potential of immunological approaches without chemotherapy in dMMR/MSI.

### Pathological complete response (pCR) and adjuvant immunotherapy

While pCR may be a surrogate survival marker for locally advanced GEA patients treated with perioperative chemotherapy [57], its prognostic role is unclear when the neoadjuvant treatment includes radiotherapy [58, 59]. The CheckMate-577 phase III trial demonstrated that adjuvant nivolumab was effective in esophageal and GEJC patients with residual disease after neoadjuvant chemoradiotherapy and surgery [60], meeting the primary endpoint (disease-free survival, DFS), but OS data is pending. The VESTIGE phase II trial evaluated the role of adjuvant nivolumab-ipilimumab *versus* FLOT in GEA patients with residual pathologic disease after having received neoadjuvant FLOT and surgery. This trial found no efficacy with adjuvant immunotherapy, but supported FLOT’s efficacy for all patients, regardless of pathological response [61].

### HER2-targeted therapy

Two non-randomized phase II trials (NEOHX and HER-FLOT) showed favorable results with trastuzumab added to perioperative chemotherapy [62, 63]. The randomized PETRARCA phase II trial [64] demonstrated improved pCR rates with trastuzumab and pertuzumab, but increased toxicity. Unfortunately, this trial was stopped prematurely, making survival outcomes unassessable. Finally, the randomized INNOVATION phase II trial, evaluating trastuzumab alone or trastuzumab-pertuzumab to perioperative chemotherapy, showed better efficacy (pCR) in the trastuzumab-only arm (toxicity limited the feasibility of double HER2-inhibition) [65]. The recently reported Survival results from INNOVATION suggested that trastuzumab may be considered only when tumor shrinkage is needed [66].

## Current pathological considerations for locally advanced resectable GEA

### Surgical pathology report

Evaluation of the neoadjuvant effect must be included in the surgical pathology report. A non-pCR result represents a new scenario for immunotherapy in the adjuvant setting [60]. For surgical specimens without grossly obvious residual tumor, the entire tumor site, including the GEJ or ulcer/tumor, should be thoroughly sampled. These specimens must then be carefully evaluated to confirm the presence or absence of residual neoplastic cells.

### MMR/MSI

Universal MSI testing by MMR IHC or polymerase chain reaction (PCR)/next-generation sequencing (NGS) is recommended for all newly diagnosed GEA patients. Testing should be performed on FFPE tissue sections (Table 2).

**Table 2:** Summary of recommendations for MSI testing in the framework of immunotherapy with comments from the ESMO Translational Research and Precision Medicine Working Group consensus panel

Recommendation A:	Immuno-histochemistry	The first choice is IHC, using antibodies recognizing the four MMR proteins: MLH1, MSH2, MSH6, and PMS2
	*Main comment:*	*MMR proteins form heterodimers and for accurate IHC interpretation, the consensus panel emphasizes the following: mutations in MLH1 are associated with IHC loss of both MLH1 and PMS2, whereas mutations in MSH2 are linked to the loss of both MSH2 and MSH6. Isolated losses of PMS2 or MSH6 have been observed, reinforcing the recommendation to use all four antibodies in testing. (*Coefficient of agreement: strong (8.7))
Recommendation B:	Polymerase chain reaction	In case of uncertainty with IHC, confirmatory molecular analysis is mandatory. The first-line molecular analysis is PCR, which can be performed using one of two recommended panels: (i) a panel with two mononucleotide repeats (BAT-25 and BAT-26) and three dinucleotide repeats (D5S346, D2S123 and D17S250) or (ii) a panel with five poly-A mononucleotide repeats (BAT-25, BAT-26, NR-21, NR-24, NR-27). The five poly-A panel is the recommended option due to its superior sensitivity and specificity
	*Main comment:*	*Both the recommended panels have been and continue to be utilized in clinical trials for assessing MSI. Molecular tests provide the highest levels of specificity and sensitivity in MSI testing. (*Coefficient of agreement: strong (8.6))
Recommendation C:	Next-generation sequencing	NGS is another molecular testing method for assessing MSI. Its main advantages lie in the ability to simultaneously analyze MSI and determine tumor mutational burden (TMB), providing a more comprehensive profile of the tumor,
	*Main comment*	*NGS should be performed only at selected centers specialized in these techniques. (*Coefficient of agreement: VERY strong (9.0))

Coefficient of agreement ranges from 0 = totally disagree, to 10 = totally agree. IHC, immunohistochemistry; MMR, mismatch repair; MSI, microsatellite instable; PCR, polymerase chain reaction; NGS, next-generation sequencing; HER2, human epidermal growth factor receptor 2

## Pathological considerations for the near future

PD-L1 determined by CPS or TAP, and/or HER2 may be future biomarkers in the setting of perioperative GEA treatment.

## Esophageal squamous cell carcinoma (ESCC)

Esophageal squamous cell carcinoma (ESCC) is the most common cause of esophageal cancer worldwide. Occurring primarily in the upper part of the esophagus, ESCC is strongly associated with poverty, alcohol, and tobacco use. The TCGA identified three subtypes of ESCC (ESCC1, ESCC2, and ESCC3), each associated with specific molecular pathway defects [10]. To date, there are no clear matched therapeutic options available for these subtypes.

## Treatment of locally advanced, unresectable, or metastatic ESCC

Patients with advanced ESCC treated with standard-of-care platinum doublet chemotherapy have a historical mOS of less than 1 year, but the introduction of anti-PD1 agents has improved outcomes. ESCC shows slightly more sensitivity to ICIs than esophageal adenocarcinoma, with greater benefit seen in tumors with elevated levels of PD-L1 expression [18].

The Phase III KEYNOTE-590 trial showed a survival benefit with pembrolizumab plus chemotherapy in patients with esophageal cancer with a PD-L1 CPS ≥ 10, regardless of histology (mOS 13.9 vs. 8.8 months; HR 0.57 [95% CI, 0.43–0.75]; *p* < 0.0001). Although this trial included both esophageal cancer histologies, the majority were ESCC (73%) [18]. The phase III CheckMate 648 trial, including only ESCC patients, showed that nivolumab plus first-line chemotherapy improved mOS, with the greatest benefit in tumors with PD-L1 TPS ≥ 1% (mOS 15.0 vs. 9.1 months; HR 0.59 [95% CI, 0.46–0.76]; *p* = 0.0001) [67]. Similarly, the RATIONALE-306 phase III study showed benefit with tislelizumab to chemotherapy in ESCC patients with PD-L1 tumor area positivity (TAP) ≥ 10% (mOS 17.2 vs. 10.6 months) (HR 0.66 [95% CI, 0.54–0.80]; *p* < 0.0001) [68]. Currently, pembrolizumab (PD-L1 CPS ≥ 10), nivolumab (PD-L1 TPS ≥ 1%), and tislelizumab (TAP ≥ 5%) are approved by EMA for use with chemotherapy.

For ESCC patients who progress after first-line treatment with good performance status (PS/ECOG), a second-line chemotherapy regimen with taxanes or irinotecan is recommended [4]. The Phase III ATTRACTION-03 trial demonstrated improved survival with nivolumab as second-line therapy compared to chemotherapy, though mainly in Asian populations [69]. Similarly, the KEYNOTE-181 Phase III trial with pembrolizumab in ESCC and esophageal adenocarcinoma patients reported positive results only in PD-L1 CPS ≥ 10 [70]. Finally, the global Phase III RATIONALE-302 trial demonstrated that tislelizumab provided improved outcomes in second-line ESCC patients with TAP ≥ 10%, supporting the above results. Median OS was 8.6 months for tislelizumab versus 6.3 months for chemotherapy (HR 0.70 [95% CI, 0.57–0.85]; one-sided *p* = 0.0001), and 10.3 months versus 6.8 months (HR 0.54 [95% CI, 0.36–0.79]; one-sided *p* = 0.0006) in patients with TAP ≥ 10% [71].

## Current pathological considerations for locally advanced unresectable or metastatic ESCC

PD-L1 expression in advanced disease is evaluated using the tumor proportion score (TPS) for nivolumab or the CPS for pembrolizumab treatment. In squamous cell carcinoma, PD-L1 expression is slightly more frequent in tumor cells than in GEA [18].

PD-L1 expression, as measured by TPS, evaluates the percentage of viable tumor cells with partial/complete membrane staining regardless intensity. A TPS ≥ 1% indicates PD-L1 positivity and guides first-line treatment decisions with nivolumab or nivolumab plus ipilimumab [67].

CPS calculation has been previously described (Current pathological considerations for locally advanced unresectable or metastatic GEA). A CPS ≥ 10 indicates PD-L1 positivity for first-line pembrolizumab treatment [70]. The CheckMate 648 study determined TPS using the PD-L1 IHC 28-8 pharmDx assay, while the KEYNOTE-590 study used the PD-L1 IHC 22C3 assay. Recent studies suggested a high concordance and potential interchangeability for PD-L1 evaluation between these assays [72, 73].

## Pathological considerations for the near future

In the RATIONALE-306 study, PD-L1 expression was assessed with the SP263 antibody (Ventana, Roche) using TAP. TAP score has been previously described (Pathological considerations for the near future of locally advanced unresectable or metastatic GEA). Patients with TAP score ≥ 10 PD-L1 were considered positive, although EMA approval is based on TAP ≥ 5 [68]. Table 3 summarizes therapeutic approaches and pathological considerations for locally advanced unresectable or metastatic ESCC.

**Table 3:** Bullet points for esophageal squamous cell carcinoma

Therapeutic approaches for locally advanced unresectable or metastatic ESCC
First-line chemotherapy combined with immunotherapy for those cases with high PD-L1 expression (. Nivolumab approved for TPS ≥ 1% and pembrolizumab approved for CPS ≥ 10). Tislelizumab has been recently approved in the second-line setting.
Biomarker determination for locally advanced unresectable or metastatic ESCC
PD-L1 assessment using TPS if treatment with nivolumab or CPS in case of pembrolizumab treatment. TAP is a novel score for PD-L1 assessment associated with emerging indications

ESCC, esophageal squamous cell carcinoma; PD-L1, programmed cell death protein 1; TPS, tumor positivity score; CPS, combined positivity score; TAP, tumor area positivity

## Treatment of locally advanced resectable ESCC

As mentioned above, the phase III CheckMate-577 trial established the adjuvant nivolumab’s role in locally advanced resectable esophageal cancer (both adenocarcinoma and ESCC) and GEJC with residual disease after neoadjuvant chemoradiotherapy and surgery. However, while the primary endpoint of DFS was met [median of 22.4 vs*.* 11.0 months for nivolumab *vs*. placebo (HR 0.69; 96.4% CI, 0.56–0.86; *p* = 0.00039)], OS data are not yet available [60]. In addition, post hoc analyses showed that DFS benefit was limited to patients with PD-L1 CPS ≥ 5 tumors.

## Pathological considerations for locally advanced resectable ESCC

As with GEA, the evaluation of the neoadjuvant therapy effect in the operated ESCC patients should be included in the surgical pathological report (http://www.cap.org).

## Next-generation sequencing (NGS) and liquid biopsy

While NGS is increasingly being used to select targeted therapies in many tumors, its role in GEA remains undefined in routine clinical practice. Currently, biomarkers in GEA and ESCC are identified through IHC or in situ hybridization (ISH), with no standard-of-care applications for NGS. However, NGS is actively used in research to identify response markers, and rare molecular subsets for new therapies, such as agnostic drugs or clinical trials. Liquid biopsy primarily refers to circulating tumor DNA (ctDNA), but also includes circulating tumor cells, non-coding RNAs, exosomes, and proteins. This minimally invasive method provides insights into tumor heterogeneity over time. While most studies focus on blood samples, non-blood fluids (saliva, gastric juice, urine, stool, and ascitic fluid) are also potential biomarker sources. Future applications include early cancer detection, monitoring molecular residual disease (MRD), early detection of recurrence, and molecular characterization of treatment response and resistance [74].

## Sampling issues

### Spatial and temporal heterogeneity in gastroesophageal cancer

The high mortality of GEC is largely attributed to late-stage detection and the inadequate responses to existing therapies. A significant factor contributing to the poor clinical outcomes of target therapies is the disease’s highly heterogeneous nature and variability in biomarkers expression. Notably, HER2 expression shows both spatial and temporal heterogeneity, a pattern also observed with PD-L1 and CLDN18.2.

Spatial heterogeneity involves different tumor subclones existing in various anatomical locations, which may hinder reliable biomarker assessment, especially in small endoscopic samples often the only collected sample from metastatic patients. According to the literature, 69–75% of the cases exhibit spatial heterogeneity in HER2 expression [75, 76]. To address this issue, guidelines recommend obtaining 6 to 8 biopsy fragments from viable tumors [30]. In GC, PD-L1 expression frequently shows significant discordance between biopsy and surgically resected tissues [33], requiring at least five GC biopsy samples for accurate diagnosis. [77]. In addition, approximately one third of cases display notable intratumor variability in CLDN18.2 expression, highlighting the need for at least six biopsies from the primary tumor to effectively tackle intratumor heterogeneity [41, 78].

Another challenge associated with heterogeneity is the discrepancy in biomarker expression between primary tumors and metastatic sites. While HER2 showed over 93% concordance by both IHC and FISH between primary tumor and paired metastatic sites, in patients who had not received anti-HER2 drugs [79], the large study by Park et al., showed that 5.7% of metastases from initially HER2-negative tumors were HER2-positive, predominantly in liver metastases [80]. In contrast, PD-L1 testing revealed only a 61% concordance rate, indicating a significant spatial heterogeneity between primary tumors and metastases [33]. Regarding CLDN18.2 positivity, 25.2% of patients exhibited discordant results between primary and metastatic sites, with varying positivity rates across different metastatic organs (peritoneal lesions showed the highest positivity rate and liver lesions the lowest) [81].

In addition, temporal heterogeneity secondary to tumor clonality has been noted, with up to 30% of GEA cases loosing HER2 expression during progression to a trastuzumab-based first line [36]. This requires reassessing HER2 status for prescribing T-Dxd in the second lines of therapy [82–84]. Furthermore, 57–63% concordance rate has also been reported for tumors before and after chemotherapy [33].

Consequently, global recommendations emphasize the importance of the re-evaluating biomarkers in metastatic tissue whenever possible, especially when temporal heterogeneity is suspected [36] (Table 4).

**Table 4:** Recommendations for biomarker testing

Samples	Requirement
Primary tumor	Minimum 6 biopsies, if possible
Synchronous carcinomas	Test all carcinomas
Metachronous carcinomas	Test all carcinomas
Metastatic carcinomas	Test it (if available)
Metachronous metastatic carcinoma	Test the most recent carcinoma (if available)

## Biomarker sequencing and co-expression analysis for treatment decisions

Testing multiple biomarkers is essential in metastatic GEA; however, it can be challenging with limited tumor sample, potential delaying chemotherapy initiation. To avoid delays, upfront and parallel testing for key actionable biomarkers with a 7-days turnaround is critical for timely therapeutic decisions [85, 86].

Given the limited scientific data in GEA, clinical practice should consider benefits, toxicity profiles, and patient preferences when managing cases with biomarker co-expression. Immunotherapy should be offered for dMMR/MSI cases, regardless of other biomarkers, while HER2-positive and PD-L1 high GEA patients should receive anti-HER2 and anti-PD1 agents. Finally, CLDN18.2-positive and high PD-L1 GEA cases may be treated based on PD-L1 thresholds: cases with PD-L1 CPS ≥ 10 are suitable for immunotherapy, while those with PD-L1 CPS < 5 may require alternative strategies (Fig. 1).

## Data Availability

Not applicable.
