# Correction: Biomarkers in gastroesophageal cancer 2025: an updated consensus statement by the Spanish Society of Medical Oncology (SEOM) and the Spanish Society of Pathology (SEAP)

**DOI:** 10.1007/s12094-025-03941-x

**Published:** 2025-06-28

**Authors:** Maria Alsina Maqueda, Ana Teijo Quintáns, Miriam Cuatrecasas, Maria Jesús Fernández Aceñero, Ana Fernández Montes, Carlos Gómez Martín, Paula Jiménez Fonseca, Carolina Martínez Ciarpaglini, Fernando Rivera Herrero, Mar Iglesias Coma

**Affiliations:** 1 Medical Oncology Department, Unidad de Oncología Médica Traslacional, Hospital Universitario de Navarra, Navarrabiomed -Instituto de Investigación Sanitaria de Navarra, Pamplona, Spain; 2 Pathology Department, Gastrointestinal and Neuroendocrine Tumors Research Group, Hospital Universitario 12 de Octubre, Research Institute (Imas12), Madrid, Spain; 3 Pathology Department, Hospital Clinic de Barcelona, Biomedical Research Institute IDIBAPS, University of Barcelona, Barcelona, Spain; 4 Department of Legal Medicine, Psychiatry and Pathology, Surgical Pathology Department, Hospital Clínico Universitario San Carlos, Instituto de Investigación Sanitaria Clínico San Carlos (IdISSC), Universidad Complutense, Madrid, Spain; 5 Madrid, Spain; 6 Medical Oncology Department, Complexo Hospitalario Universitario de Ourense, Ourense, Spain; 7 Gastrointestinal Cancer and Early Clinical-Translational Research Units, Medical Oncology Division, 12 de Octubre University Hospital, Madrid, Spain; 8 Department of Medical Oncology, Hospital Universitario Central de Asturias, ISPA, Oviedo, Spain; 9 Pathology Department, Hospital Clínico Universitario de Valencia, Biomedical Research Institute INCLIVA, University of Valencia, Valencia, Spain; 10 Medical Oncology Department, Hospital Universitario Marqués de Valdecilla, IDIVAL, Santander, Spain; 11 Pathology Department, Hospital del Mar, Pompeu Fabra University, Hospital del Mar Research Institute, Barcelona, Spain

**Correction: Clinical and Translational Oncology** 10.1007/s12094-025-03865-6

In this article the affiliation details for Author Maria Alsina Maqueda were incorrectly given as “Medical Oncology Department, Unidad de Oncología Médica Traslacional, Hospital Universitario de Navarra, Navarrabiomed-Centro de Investigación Sanitaria de Navarra, Pamplona, Spain” but should have been “Medical Oncology Department, Unidad de Oncología Médica Traslacional, Hospital Universitario de Navarra, Navarrabiomed-Instituto de Investigación Sanitaria de Navarra, Pamplona, Spain”.

The reference citation displayed in Fig. 1 caption was incorrectly given as “(adapted from https://doi.org/10.1016/j.esmoop.2023.102226)” but should have been “(adapted from 10.1016/j.esmogo.2024.100086)”.

In the sentence beginning “Fluoropyrimidine and platinum combination therapy is the standard first-line treatment…”, the reference citation “[11]” should have read “[6]”.

In the section “Current pathological considerations for locally advanced unresectable or metastatic GEA”, the equation was displayed incorrectly as,

$${\text{CPS}} = \frac{{{\text{Number of positive tumor cells}} + {\text{Number of mononucleated immune cells}} \times 100}}{{\text{Total viable tumor cells}}} = 0 - 100.$$

but it should have been,

$${\text{CPS}} = \frac{{{\text{Number of PD-L1 positive tumor cells}} + {\text{Number of PD-L1 positive mononuclear inflammatory cells}}}}{{\text{Total tumor cells}}} \times 100 = 0 - 100.$$

In the sentence beginning “The final cut-off for targeted therapy needs…”, the reference citation “[41]” should have read “[26]”.

In the sentence beginning “A meta-analysis of phase III trials, including perioperative chemotherapy…”, the reference “[50]” should instead have referred to “[51]”. The complete details of this reference should have been “51. Pietrantonio F, Miceli R, Raimondi A, Kim YW, Kang WK, Langley RE, et al. Individual Patient Data Meta-Analysis of the Value of Microsatellite Instability As a Biomarker in Gastric Cancer. J Clin Oncol. 2019 Dec 10;37(35):3392–3400. 10.1200/JCO.19.01124. Epub 2019 Sep 12. PMID: 31513484.”

In the sentence beginning “Nevertheless, the results of the randomized phase…” a new reference should have been included as “Nevertheless, the results of the randomized phase II DANTE trial with FLOT regimen suggested potential efficacy of taxanes-containing regimens in this setting [54]”. The complete details of this reference should have been “54. Lorenzen S, Götze TO, Thuss-Patience P, Biebl M, Homann N, Schenk M, et al. Perioperative Atezolizumab Plus Fluorouracil, Leucovorin, Oxaliplatin, and Docetaxel for Resectable Esophagogastric Cancer: Interim Results From the Randomized, Multicenter, Phase II/III DANTE/IKF-s633 Trial. J Clin Oncol. 2024 Feb 1;42(4):410–420. 10.1200/JCO.23.00975. Epub 2023 Nov 14. PMID: 37963317.”

The sentence “However, adding durvalumab to the FLOT regimen increased pathologic complete response (pCR) rates, though survival data are pending [53]” was incorrect and should have been “However, adding durvalumab to the FLOT regimen increased pathologic complete response (pCR) rates, and also event free survival outcomes, according to a recent press-release [52].”

In the sentence beginning “ESCC shows slightly more sensitivity to ICIs than esophageal adenocarcinoma…”, the reference citation “[34]” should have read “[18]”.

The old reference “53. Janjigian YY, Al-Batran SE, Wainberg ZA, Van Cutsem E, Molena D, Muro K, et al. Pathological complete response (pCR) to 5-fluorouracil, leucovorin, oxaliplatin and docetaxel (FLOT) with or without durvalumab (D) in resectable gastric and gastroesophageal junction cancer (GC/GEJC): subgroup analysis by region from the phase 3, randomized, double-blind MATTERHORN study. JCO [Internet]. 2024;42(3_suppl):LBA246. 10.1200/JCO.2024.42.3_suppl.LBA246.was incorrect and it should be eliminated.

References 8 and 10 were incorrect and should have been interchanged in the References section. Due to the insertion of new Reference 51, the subsequent references are renumbered till the end of the article. References 58 and 60 were incorrect and the correct details for the renumbered References 59 and 61 are as follows:

**Incorrect references:**

8. Cancer Genome Atlas Research Network. Comprehensive molecular characterization of gastric adenocarcinoma. Nature. 2014;513(7517):202–9.
10. Cancer Genome Atlas Research Network, Analysis Working Group: Asan University, BC Cancer Agency, Brigham and Women’s Hospital, Broad Institute, Brown University, et al. Integrated genomic characterization of oesophageal carcinoma. Nature. 2017;541(7636):169–75.
58. van Hagen P, Hulshof MCCM, van Lanschot JJB, Steyerberg EW, van Berge Henegouwen MI, Wijnhoven BPL, et al. Preoperative chemoradiotherapy for esophageal or junctional cancer. N Engl J Med. 2012;366(22):2074–84.
60. Smyth E, Mauer M, Cella C, Ben-Aharon I, Piessen G, Wyrwicz L, et al. O-6 EORTC 1707 VESTIGE: adjuvant immunotherapy in patients (pts) with resected gastroesophageal adenocarcinoma (GEA) following preoperative chemotherapy with high risk for recurrence (ypN + and/or R1)—an open-label randomized controlled phase II study. Ann Oncol [Internet]. 2023;34:S182–3.

**Correct references:**

8. Cancer Genome Atlas Research Network, Analysis Working Group: Asan University, BC Cancer Agency, Brigham and Women’s Hospital, Broad Institute, Brown University, et al. Integrated genomic characterization of oesophageal carcinoma. Nature. 2017;541(7636):169–75.
10. Cancer Genome Atlas Research Network. Comprehensive molecular characterization of gastric adenocarcinoma. Nature. 2014;513(7517):202–9.
51. Pietrantonio F, Miceli R, Raimondi A, Kim YW, Kang WK, Langley RE, et al. Individual Patient Data Meta-Analysis of the Value of Microsatellite Instability As a Biomarker in Gastric Cancer. J Clin Oncol. 2019 Dec 10;37(35):3392–3400. 10.1200/JCO.19.01124. Epub 2019 Sep 12. PMID: 31513484.
52. Janjigian YY, Al-Batran SE, Wainberg ZA, Van Cutsem E, Molena D, Muro K, et al. LBA73 pathological complete response (pCR) to durvalumab plus 5-fluorouracil, leucovorin, oxaliplatin and docetaxel (FLOT) in resectable gastric and gastroesophageal junction cancer (GC/GEJC): Interim results of the global, phase III MATTERHORN study. Ann Oncol [Internet]. 2023;34:S1315–6.
53. Shitara K, Rha SY, Wyrwicz LS, Oshima T, Karaseva N, Osipov M, et al. Neoadjuvant and adjuvant pembrolizumab plus chemotherapy in locally advanced gastric or gastro-oesophageal cancer (KEYNOTE-585): an interim analysis of the multicentre, double-blind, randomised phase 3 study. Lancet Oncol. 2024;25(2):212–24.
54. Lorenzen S, Gotze TO, Thuss-Patience P, Biebl M, Homann N, Schenk M, et al. Perioperative Atezolizumab Plus Fluorouracil, Leucovorin, Oxaliplatin, and Docetaxel for Resectable Esophagogastric Cancer: Interim Results From the Randomized, Multicenter, Phase II/III DANTE/IKF-s633 Trial. J Clin Oncol. 2024 Feb 1;42(4):410–420. 10.1200/JCO.23.00975. Epub 2023 Nov 14. PMID: 37963317.
55. André T, Tougeron D, Piessen G, de la Fouchardière C, Louvet C, Adenis A, et al. Neoadjuvant nivolumab plus ipilimumab and adjuvant nivolumab in localized deficient mismatch repair/ microsatellite instability-high gastric or esophagogastric junction adenocarcinoma: the GERCOR NEONIPIGA phase II study. J Clin Oncol. 2023;41(2):255–65.
56. Raimondi A, Lonardi S, Murgioni S, Cardellino GG, Tamberi S, Strippoli A, et al. Tremelimumab and durvalumab as neoadjuvant or non-operative management strategy of patients with microsatellite instability-high resectable gastric or gastroesophageal junction adenocarcinoma: the INFINITY study by GONO. Ann Oncol. 2024;S0923–7534(24):04953–6.
57. Lorenzen S, Thuss-Patience P, Al-Batran SE, Lordick F, Haller B, Schuster T, et al. Impact of pathologic complete response on disease-free survival in patients with esophagogastric adenocarcinoma receiving preoperative docetaxel-based chemotherapy. Ann Oncol. 2013;24(8):2068–73.
58. Reynolds JV, Preston SR, O’Neill B, Lowery MA, Baeksgaard L, Crosby T, et al. Trimodality therapy versus perioperative chemotherapy in the management of locally advanced adenocarcinoma of the oesophagus and oesophagogastric junction (Neo-AEGIS): an open-label, randomised, phase 3 trial. Lancet Gastroenterol Hepatol. 2023;8(11):1015–27.
59. Hoeppner J, Brunner T, Schmoor C, Bronsert P, Kulemann B, Claus R, et al. Perioperative Chemotherapy or Preoperative Chemoradiotherapy in Esophageal Cancer. N Engl J Med. 2025 Jan 23;392(4):323–335. PMID: 39842010. 10.1056/NEJMoa2409408.
60. Kelly RJ, Ajani JA, Kuzdzal J, Zander T, Van Cutsem E, Piessen G, et al. Adjuvant nivolumab in resected esophageal or gastroesophageal junction cancer. N Engl J Med. 2021;384(13):1191–203.
61. Lordick F, Mauer ME, Stocker G, Cella CA, Ben-Aharon I, Piessen G, et al. Adjuvant immunotherapy in patients with resected gastric and oesophagogastric junction cancer following preoperative chemotherapy with high risk for recurrence (ypN+ and/or R1): European Organisation of Research and Treatment of Cancer (EORTC) 1707 VESTIGE study. Ann Oncol. 2025 Feb;36(2):197–207. 10.1016/j.annonc.2024.10.829. Epub 2024 Nov 13. PMID: 39542422.

The original article has been corrected.

